# ‘I think that we can effect change’: Psychologist use of social media for social justice advocacy

**DOI:** 10.1111/papt.12601

**Published:** 2025-05-11

**Authors:** Ella White, Terry Hanley

**Affiliations:** ^1^ Manchester Institute of Education, University of Manchester Manchester UK

**Keywords:** mental health, psychologist, social justice, social media

## Abstract

**Introduction:**

Psychologists can bring social justice into their professional presence on social media, with the public perceiving health care professionals as a reputable source of information online. This study aimed to explore practitioner psychologists' use of social media for social justice advocacy as a mental health influencer.

**Methods:**

Twelve UK‐based practitioner psychologists were interviewed who had an Instagram account that they used as a mental health influencer. The semi‐structured interview transcripts were analysed using reflexive thematic analysis.

**Results:**

Systemic issues have motivated many psychologists to begin work as a mental health influencer. There are challenges between the use of social media for social justice advocacy and for business purposes to promote private practice. Psychologists can use social media to share psychoeducation to increase mental health literacy and encourage access to therapy. The accessibility of this content is particularly valuable for people from marginalised communities and for people on long waiting lists to access mental health support. Psychologists can use social media to raise critical consciousness of social inequalities and reduce an individual's sense of self‐blame.

**Conclusion:**

Irrespective of working in the public sector or private practice, there are opportunities for psychologists to use social media as a resource for social justice advocacy.

## INTRODUCTION

The term ‘social justice’ references a distributive justice stance suggesting society should provide fair treatment and an equal share of benefits, resources and opportunities to individuals and groups (Chung & Bemak, 2012). Bringing social justice into therapeutic practice is central to the values of counselling psychologists (Winter, 2019). Psychologists using a social justice model try to consider distress in the context of social factors, which could predispose psychological distress such as poverty and discrimination due to diversity factors, rather than using an individualised model of causation which may create self‐blame and guilt (Kagan et al., 2010). Goodman et al. (2004) devised six principles for psychologists to consider when incorporating social justice into their work encompassing: ongoing self‐examination, sharing power, giving voice, facilitating consciousness raising, building on strengths and leaving clients with tools to work towards social change. In the United Kingdom, although the term social justice is not directly used within the Health and Care Professions Council (Health and Care Professions Council, 2024a) standards of proficiency, components of social justice are present. Psychologists are encouraged to be aware of the impact of culture, equality and diversity on practice and to practice in a non‐discriminatory and inclusive manner. This includes the recognition and challenging of barriers to inclusion to implement change.

Al'Uqdah et al. (2019) suggest that encouraging critical thinking on social media is a form of social justice advocacy, through providing opportunities for marginalised communities to access information. This is supported by Tribe and Bell (2018) who perceive social media as a platform within which to access marginalised or ‘hard to reach’ groups, as well as providing opportunities to create links with other professionals or charities online. Psychologists use of social media can, therefore, be perceived as a way to advocate for social justice at a wider level. This fits with the principle of facilitating consciousness raising by Goodman et al. (2004), as through psychoeducation on social media psychologists can educate a wider population about the role of systemic issues such as racism, sexism and class on their psychological distress. Social media can be used to increase critical consciousness, which helps the public better understand the impact of inequalities and oppression on mental health (Freire, 1973, 1978), reducing shame by evidencing how issues are at a systemic level (Kagan et al., 2010). Equipped with this knowledge, the public is more likely to work together towards social action and implementing changes in society. Social media may be another avenue for social justice work beyond what they already do in clinical practice.

Triplett et al. (2022) define a mental health influencer as a mental health professional who uses social media to share psychoeducation and anecdotes from their personal or professional experiences. Many psychologists who use social media with a professional presence may not self‐identify as a mental health influencer, and yet match this definition due to the content they share. Mental health influencers can use social media for social justice advocacy to educate the public via social media on issues that increase political and social responsibility (Al'Uqdah et al., 2019). Myers et al. (2012) identified multiple beneficial purposes for a psychologists' professional presence on social media, including networking and information sharing with other psychologists around therapeutic work or research. Tunnecliff et al. (2015) found that 95.9% of health professional researchers believed social media could be used for recruiting participants and sharing research findings, potentially resulting in larger sample sizes using more diverse populations. Psychologists can also use social media to advertise their professional services and create a personal brand (Cederberg, 2017). The Health and Care Professions Council (2024b) states that social media can be used in a professional capacity to help the public understand what the practitioner does and raise the profile of their profession.

The growing number of followers of mental health influencers may be representative of the public perception of mental health shifting to be more receptive to learning about different types of psychological distress. Ofcom (2023) reported that 78% of people had used the internet, defined as social media apps and websites, to support their wellbeing, such as looking up ways to relax. Additionally, the high following may be indicative of societal changes, with this study conducted during the COVID‐19 pandemic and a cost of living crisis; thus, people may be searching for additional support with their mental health online (Pretorius et al., 2019). The COVID‐19 pandemic saw a rise in follower counts of health care professionals, as many people feared for their mental and physical health and wanted information from a trustworthy, reputable source (Furstrand et al., 2021). Medina and Montell (2022) queried if other factors such as climate change and untrustworthy governments also contributed to people turning to therapists on social media for support with their anxiety about these issues.

A systematic review of 34 studies found young people aged 13 to 18 years believed mental and physical health information shared by health care professionals on social media was more trustworthy (Freeman et al., 2023). Young people trusted health information more when shared by respected organisations, such as the NHS or government. Sharing mental health campaigns on social media significantly increases campaign engagement, with the benefits of being low‐cost with high scalability (Latha et al., 2020). Additionally, social media mental health campaigns have proven an effective way to reach communities where mental health support may be less accessible, such as ethnic minorities and lower socioeconomic groups (Alonzo & Popescu, 2021). Naslund et al. (2020) suggest social media could be used to enhance existing mental health services and connect people to supportive peers. Therefore, mental health influencers can use social media to promote multiple mental health resources, including mental health campaigns, evidence‐based health information and access to support services.

### Rationale and research question

Public interest in accessing mental health information online has increased, with social media providing a platform for practitioners to use their knowledge and professional status as a reputable source of information. Much of the literature around psychologists' use of social media is focused on ethical issues that the psychologist may encounter online, rather than their purpose for using social media as a psychologist. The broader research project (White & Hanley, 2025) explored psychologists' experiences of social media use as a mental health influencer. This study employed a qualitative design to interview psychologists who hold an Instagram account as a mental health influencer, to gain a detailed understanding of their experiences. Part of the interview schedule was focused on social justice advocacy, which became a larger theme than expected during the interviews and analysis. Currently, there is no qualitative literature exploring psychologists' perspectives on using their professional presence on social media for social justice advocacy. Based on this rationale, the following research question was explored in this current paper: what are practitioner psychologists' experiences of Instagram use for social justice advocacy as mental health influencers?

## METHODOLOGY

### Design and procedure

An inductive qualitative research design was chosen as it is hermeneutic and phenomenological to centre the perspectives and experiences of the participants (Braun & Clarke, 2013; Howitt, 2019). Reflexive thematic analysis was used, with a ‘Big Q'’ approach prizing subjectivity over ‘small q'’ values of replicability and reliability (Clarke & Braun, 2018; Kidder & Fine, 1987). This research is situated within a social constructionist epistemological stance, with the belief that knowledge is constructed within a social and cultural context, with the meaning attached subject to change (Burr, 2015). From a relativist ontological stance, the use of a qualitative approach provides the opportunity to understand how psychologists interpret their own reality as a mental health influencer on Instagram (Baghramian, 2019).

This study received full ethical approval from the University of Manchester. The study was reviewed at the department level due to the low‐risk of interviewing psychologists over the age of 18 and not involving sensitive information. The project adhered to the ethical requirements of the Health Care and Professions Council (2016) and British Psychology Society (2021).

After expressing interest in the study, participants were emailed the participant information sheet and consent form to sign before the interview. The interviews were conducted remotely via Zoom to remove accessibility barriers, as participants were located in multiple areas of the United Kingdom and had minimal time available for participation alongside their work (Afzalan & Muller, 2018; Deakin & Wakefield, 2013).

Individual semi‐structured interviews were used, due to their flexibility to explore new ideas raised by participants, whilst still retaining a level of consistency through the interview schedule (Braun & Clarke, 2006). The interview schedule comprised 11 open questions to elicit rich data through as personal a response to the questions as possible (Willig & Rogers, 2017). The interviews lasted between 30 min and 1 h and 20 min, with the average interview time being 1 h and 5 min. A debrief sheet was emailed after the interview detailing available support services and the study rationale.

### Participants

The inclusion criteria are UK‐based qualified or trainee practitioner psychologists with a minimum of 1000 Instagram followers. Participants were recruited through purposive homogenous sampling, regarded as an effective sampling method to recruit available participants from a target population (Patton, 2002). Psychologists were directly messaged on Instagram from an account created for this purpose using the same initial recruitment message for all prospective participants. Participants were found by typing ‘psychologist’ into the search function of Instagram and reviewing the comments and followers of psychologist's profiles for further psychologists to contact. One participant was recruited through snowball sampling (Goodman, 1961).

Twelve participants were interviewed over a three‐month period from October 2022 to January 2023. Table 1 details participant demographic information, with 75% of participants being clinical psychologists and only one participant identifying as male (8%).

**TABLE 1:** Participant demographic information.

Participant pseudonym	Age	Gender	Ethnicity	Profession	Service
Bella	41	Female	White British	Clinical psychologist	Private practice
Echo	45	Female	White British	Clinical psychologist	Third sector
Evie	36	Female	White British	Clinical psychologist	Private practice
Grace	41	Female	White British	Clinical psychologist	Private practice
Ivy	35	Female	White British	Clinical psychologist	Third sector
Laura	36	Female	White British	Clinical psychologist	NHS
Lily	34	Female	White British	Counselling psychologist	Private practice
Liz	43	Female	White British	Clinical psychologist	Private practice
Louise	45	Female	White British	Clinical psychologist	Private practice
Mia	41	Female	Mixed background	Clinical psychologist	Private practice
Mitch	38	Male	White British	Clinical and forensic psychologist	NHS and HMP
Sophia	29	Female	White Asian	Sport and exercise psychologist	Private practice

Participants were asked if they would like to choose their own pseudonyms. In doing so, this acknowledges the inevitable power dynamics between researcher and participant and attempts to increase collaboration where possible (Dill & Kohlman, 2014; Mertens, 2015). Many participants commented that the ability to choose their own pseudonyms felt novel and unexpected.

### Analysis

The six recursive phases of reflexive thematic analysis outlined by Braun and Clarke (2021a) were used. The primary researcher (Dr Ella White) familiarised themselves with the data through conducting and manually transcribing the interviews. White systematically read each transcript, highlighting sections and adding ‘pithy’ labels to summarise key points that may answer the research question, generating 176 codes on NVivo (Clarke & Braun, 2013). Semantic coding was utilised based on the surface meaning of the data from the participant and used key words from participants where possible when naming codes (Byrne, 2022). Codes that shared a similar underlying concept were collapsed into the same overarching code, generating 19 final codes. The collapsed codes were configured into a concept map for clarity of the relationship between codes, then organised into candidate themes using mind maps. Candidate themes and subthemes were reviewed with the second author to check they made sense in relation to the codes, original transcript and research question. The researcher considered the story of each theme to create illuminating names, drawing upon ‘punchy’ extracts of participant quotes (Braun & Clarke, 2021b; Clarke & Braun, 2013). Braun and Clarke (2021a) outline writing a brief synopsis of candidate themes, which were emailed to participants to provide member reflections. Participants' feedback was applied through a recursive process, which included changing some of the subtheme names and adding more to the story of positive experiences of social media use. The primary researcher returned to the analysis when considering the new research question for this paper focused on social justice advocacy.

### Trustworthiness and reflexivity

This research was conducted in line with guidelines for qualitative research provided by Braun and Clarke's (2006, 2021b) 15‐point thematic analysis checklist. The research was grounded in examples to illustrate the reflexive thematic analysis and the primary researcher's subsequent understanding, including transcript excerpts. The use of NVivo 12 increases the reputability of this research, with an audit trail of each version of the codes and themes generated. Member reflections were conducted for collaboration and reflexive elaboration, thus enhancing the credibility criteria of the quality of the qualitative research (Kornbluh, 2015; Tracy, 2010). Member reflections fit the relativist ontological positioning of this study, with both the voice of the participant and the researcher present acknowledging there is no one true reality (Sage, 2022).

Elliott et al. (1999) list owning one's own perspective as a key guideline for qualitative research. The primary researcher is an HCPC registered Counselling Psychologist who completed this research as part of their doctoral thesis. They have viewed a large number of Instagram posts from mental health influencers, which inspired this study as they questioned the ethics of the growing use of social media by practitioners and felt this was a gap in their professional training. Their personal experiences may have provided them with biases around practitioner social media use which they strived to make explicit within a reflexive journal during data collection and analysis. The researcher was aware of their biases as a Counselling Psychologist, reflected in their choice to include in the broader research project (White & Hanley, 2025) a subtheme on social justice which is a core value of the counselling psychology identity (Winter, 2019). The researcher felt the importance of these social justice advocacy findings should be published as a separate paper to emphasise the importance and novelty of this work. The second author is an HCPC registered Counselling Psychologist interested in the interface between therapy and technology.

## RESULTS AND DISCUSSION

Three themes were developed, each with four subthemes, which are discussed in White and Hanley (2025). The themes and subthemes associated with the broader research project can be viewed in Appendix A. All themes and subthemes were named after direct participant quotes. In summary, the first theme ‘I'm a psychologist, but I'm not your psychologist’ explored psychologists' experiences of trying to input boundaries on Instagram around self‐disclosure, risk management, time management and client work. The second theme ‘anxiety about not wanting to do the wrong thing’ highlighted the impact of an influencer's presence on psychologists' own mental health. The third theme ‘I share what I think is going to be helpful for other people and myself’ considered the psychologists' motivations for Instagram use for social justice advocacy and business promotion, and the contradictions between these.

To add richness to the discourse around how psychologists use social media as mental health influencers, the present paper is focused on how Instagram is currently being used for social justice advocacy. The findings are divided into how systemic issues are informing the psychologists' decisions to become mental health influencers, the contradictions between the use of social media for both social justice advocacy and marketing their paid services and the use of social media to raise critical consciousness and increase mental health literacy. These findings are discussed in the context of the current literature.

### Systemic issues informing decisions to become a mental health influencer

The majority of the psychologists (8/12) interviewed in this study had left the NHS or third sector and now worked in private practice. For many, this raised strong feelings of guilt that their work was now less accessible due to the cost barrier. This fits with the psychologist Gilderthrop (2021) reflecting on their decision to leave the NHS for private practice, feeling they were taking the ‘selfish route’ and ‘stepping into the dark side’. The psychologists in the present study felt selfish for leaving the NHS as if this indicated they valued money more than providing accessible care. Nonetheless, the psychologists were aware that their work was needed to provide an income for themselves and their families. Gilderthrop (2021) surveyed 47 psychologists who had left the NHS for private practice, finding the key reasons to leave included the impact on the psychologists' own mental health and physical health, with burnout and a lack of support from supervisors and managers who did not make changes to safeguard staff. The psychologists felt the demands from services were too great, with high exposure to risk combined with large caseloads due to understaffing. Some psychologists struggled with NHS service restructuring due to changing governments and funding. Another key reason to leave was the NHS no longer aligned with their values, with psychologists feeling they were pathologising people, lacking time to create thorough formulations or enough sessions to properly help clients. This fits with the psychologists in the present study (White & Hanley, 2025), with Mia describing how ‘it brings up a lot of feelings of rage’ and ‘I definitely felt much more defeated this year’ that changes to mental health services have increased the difficulty of accessing support and reduced the quality of this support in the NHS, contributing to their motivations to begin private practice.

This is further supported by Ryan et al. (2019) who used a mixed‐methods approach to understand why therapists chose to leave the NHS. The responses were categorised into three themes: undervalued profession, detrimental to wellbeing and failing service structure. The theme ‘undervalued profession’ included the NHS having limited job opportunities and a reliance on volunteers, therapists feeling disrespected and undervalued and losing paid work due to funding cuts. ‘Detrimental to wellbeing’ included feeling ethically compromised to meet service expectations at the expense of client care, and feeling the work was detrimental to their own mental health with stress and burnout. ‘Failing service structure’ comprised restrictions on how to practice and unsupportive management. Ryan et al. (2019) also surveyed therapists still employed by the NHS to understand their perceptions, with over half of therapists rating waiting times and number of sessions to be completely or mostly inadequate. Moreover, over half the therapists rated staff morale as completely or mostly inadequate. This fits with the psychologists in this study reflecting on how they had experienced burnout in their previous roles, which led them to set up private practice and a mental health influencer Instagram account. Mia was concerned that psychologists feeling unfulfilled in the NHS would use Instagram for validation: ‘I think lots of psychologists and therapists feeling very invalidated in their roles [are] turning to social media because it does give you that sense of external validation, but while not feeling ok themselves’. However, Grace described how ‘I take my time when I write posts and really consider who is this for? Is this for me? Is it for other people? Who is it benefitting?’ to reduce the risk of posting for an ego boost.

Entering private practice appeared to act as a motivator for the psychologists to set up their mental health influencer Instagram accounts on two levels. Firstly, the accounts acted as a way to assuage their guilt at leaving the NHS or third‐sector work by offering an alternative way to support the public by sharing their psychological knowledge online. In this way, the psychologists could access a much larger population than they could through one‐to‐one therapy and with no financial cost (unless further psychoeducation was behind a paywall). Secondly, some of the psychologists used their Instagram as a marketing tool to promote their therapeutic services, with Liz explaining ‘I definitely think it's a huge tool in me getting work’. As the psychologists need to find their own clients for their private practice work, Instagram offers a new way to advertise their services and help the client to gain a sense of the psychologist. Some of the psychologists reflected that this helps build trust faster and strengthen the therapeutic relationship, as the client already knows information about their lives, such as if they are a parent, and what their key values are. Evie commented that ‘[I'm] very transparent on social media. This is who I am so that the person knows what they're coming for before they start, because private work is really expensive and you want to get a good fit straight away ideally’. Hund (2023) states that ‘the influencer industry's core business is reassessing, redefining, and revaluing authenticity’ (p. 13), and so the psychologist acting authentically on social media will attract like‐minded potential clients. Therefore, the rise in private practice runs in parallel to the rise in mental health influencers. This is evidenced by Pretorius et al. (2022) reporting that 28.18% of mental health influencers' posts on Instagram were to promote their therapeutic services.

Some of the psychologists felt concerned that the NHS was monitoring their posts, which motivated them to leave for private practice. Laura shared ‘I've had my employer ask me to delete certain posts because they haven't felt appropriate’ with confusion around this subjectivity and ‘anxiety […] as to how this is going to be received’. The psychologists were cautious in what they posted for fear of negative repercussions from their employers. Sophia felt ‘paranoia’ and ‘scrutiny from other practitioners’ fearing she would be reported to the HCPC which could place their job at risk. Lily masked their identity ‘to make it that bit harder for [employers] to find me’. It feels important to note the majority of the psychologists' content was simple evidence‐based psychoeducation to contextualise how high their anxiety was around their social media presence impacting their offline work.

The psychologists were frustrated to be part of a system that advised them to be apolitical, when the NHS itself and working as a psychologist supporting people with their mental health in response to systemic issues is inherently political. Many psychologists wanted to use their accounts for social justice advocacy in an informer role sharing content to increase knowledge of the impact of systemic issues on psychological distress (Al'Uqdah et al., 2019). Mia explained how she uses her platform ‘as a space to share the kind of more systemic impact of […] what's going on politically and financially and the impact that has on mental health’. However, they believed this conflicted with NHS social media policy guidance to not post political content. Some psychologists felt so passionately about sharing social justice content that they would risk losing their NHS jobs or setting up their own private practice where they would not feel censored. Evie shared how ‘I do worry about you know getting pulled up. But I think I would defend myself, and I would know that deep down I'm staying true to my values and my beliefs, and if I lost my job then I lost my job’.

### Contradictions between social justice advocacy and marketing

There appeared to be clear tensions and contradictions between the use of social media for social justice advocacy and for business purposes that furthered the psychologists' anxieties and uncertainties. This was clearly summarised in the theme name ‘I share what I think is going to be helpful for other people and myself’ (White & Hanley, 2025). This highlighted the dichotomy between motivations to use social media to support others through sharing free accessible psychoeducation, and for the self to promote their business. One key example of this contradiction was the psychologists' confusion around where to input a paywall for their content, recognising they could raise an individual's awareness of an issue but then instigate a paywall so they cannot then access support. Questions were raised on the relationship between the quality of content and the cost, with Sophia querying ‘what's the difference between the stuff that I will put behind a paywall and like the quality of that information?’. This is particularly important to consider in the context of the cost of living crisis, combined with the power the psychologist holds with their Dr. title to influence people to purchase their recommendations.

This tension is further illustrated by the psychologists' decisions to leave the NHS to become a mental health influencer, with the psychologists referencing their guilt at no longer providing accessible therapy. Lily discussed how providing free psychoeducation resources feels particularly important when she is not working in the NHS: ‘actually right now it's really hard to get access to anything, and so if I'm able to offer something that might be useful that somebody can take away […] to try and help as many people as I can, particularly having moved into private practice, that feels really important to me’. However, the psychologists were actually able to use this account for multiple purposes, as their free posts would attract new clients for their private practice and motivate people to purchase passive products, thus also benefitting the psychologist. Therefore, even though social media use for social justice advocacy may benefit the general public, it also benefits the psychologist whether financially or through validation from likes and comments linking to the subtheme ‘it can make you feel like you're useful as a psychologist and also as a person’ (White & Hanley, 2025).

Some psychologists explained they outsourced the creation and marketing of posts to a professional without mental health training, so they could focus on their therapy work as outlined in the subtheme ‘I didn't train as a marketer or social media expert’. Whilst Bella described how her marketing skills were self‐taught and how ‘I'm now really proud to say I'm a businesswoman’, Liz comparatively explained ‘I want to be doing the stuff that's going to bring me money, I don't want to spend the whole time doing marketing stuff. I'm not a marketer, I'm a therapist. That's what I've trained to do. So I'll pay someone else to help me with some of that’. This suggests the primary purpose of these accounts may be for business promotion, rather than social justice advocacy, as the psychologist is not prioritising creating this content themselves, which casts doubt on the importance of their professional title and authenticity.

### Ways to use social Media for social justice advocacy

The three main reasons identified in this study for why practitioner psychologists used Instagram as a mental health influencer are: business purposes to promote their private therapy or passive products such as books or online courses; psychoeducation to increase knowledge of mental health and self‐care strategies which could reduce stigma and promote earlier access to therapeutic support; and social justice purposes to increase knowledge of the impact of systemic issues on mental health. There is some overlap between these purposes, such as sharing free psychoeducation which can be widely accessed by marginalised communities fitting as social justice work. The subtheme ‘I think that we can effect change’ by White and Hanley (2025) focused on psychologists' motivations to use their professional presence on social media for sharing social justice content. The ways in which psychologists could use social media for social justice advocacy as part of their practice appear to be in two main areas: raising critical consciousness and increasing mental health literacy. These two purposes will be explored in greater depth and linked to relevant literature below.

### Raising critical consciousness

The psychologists believed it was important to raise awareness on Instagram of the impact of systemic issues on mental health, to the extent that some psychologists would even risk losing their jobs in the NHS. The psychologists believed their professional training enabled them to make links between systemic issues and mental health. Mia aimed to share this knowledge to reduce self‐blame and shift thinking from ‘I thought this was my problem and now I realise that it's a systemic problem, like it's not just me’. Ivy shared how this felt empowering and in line with her values, explaining ‘I think one way of affecting change is by kind of maybe highlighting some of the issues, and getting us to zoom out and think about the wider context’. This fits with the definition of critical consciousness by Freire (1973, 1978) to facilitate a greater understanding of inequalities and oppression, which can then support mobilisation of social action and changes. Goodman et al. (2004) acknowledge that psychologists are capable of identifying systemic influences on wellbeing but are less skilled at working at the systemic level in ‘redressing those very systems and structures from which individual and community difficulties originate’ (p. 797). As this paper was published 20 years ago, social media has significantly increased in prevalence since then and so can be considered as a tool for psychologists to work at a systemic level. This could be done by using social media to raise critical consciousness by providing free accessible resources that educate individuals on the impact of systemic issues on their mental health so communities can collectively push for societal changes.

Evie conceded that Instagram was a way to reach many more people than they could be working with individual clients in therapy: ‘I think targeting people in individual therapy is great but I think there's, you know, psychological knowledge and psychoeducation that can be shared more globally and helping to improve wellbeing too’. Many psychologists primarily work with social justice at a micro level by supporting individuals with managing their psychological distress and recognising the impact of systemic issues on their distress within their formulation (Vera & Speight, 2003). However, Goodman et al. (2004) suggest this is insufficient as without macro‐level changes to society this individual‐level therapeutic work will only ever be partially successful as individuals are still situated in these social and political contexts. This epitomises the concept of the personal is political (Hanisch, 1969), with Prilleltensky (1999, p. 106) explaining that psychological distress is ‘connected to people's social support, employment status, housing conditions, history of discrimination, and overall personal and political power’ and so ‘promoting complete health means promoting social justice for there cannot be health in the absence of justice’. Therefore, this signifies the value of the use of social media as a tool for social justice advocacy at a more macro level to raise critical consciousness.

The Marmot Review (2010) declared health inequalities are a matter of social justice, with social and economic differences in health status reflective of social and economic societal inequalities. The review reported multiple health inequalities such as shorter life expectancy, shorter health expectancy and increased likelihood of both common and severe mental health conditions across social and demographic factors, such as social class, education level and ethnicity. A few psychologists referenced their sense of ‘defeat’ and ‘hopelessness’ in trying to support people with their mental health during a time when social inequalities are becoming more pronounced, due to issues such as the COVID‐19 pandemic and the cost of living crisis. The psychologists described their frustrations with the inability to signpost to support services as many services are overwhelmed, rendering their Instagram highlights around signposting less practical. This may potentially lead more people to directly message the psychologists for support as they cannot access the signposted services, increasing feelings of anxiety and burnout in the psychologists.

Al'Uqdah et al. (2019) conceptualised five roles that counselling psychologists can adopt to promote social justice on social media: the researcher, the informer, the advocator, the agitator and the supporter. The researcher uses social media to recruit participants for research and to share research findings to be more accessible to the public. The researcher may also act as a ‘lurker’ observing comments and posts by people from diverse communities with differing opinions to broaden the psychologist's understanding of an issue and inform research questions. The informer uses social media to raise awareness of social justice issues, including sharing information about social justice activities, such as an upcoming protest, or following and sharing posts by organisations who work towards improving factors that contribute to mental health. In this way, the psychologist increases their knowledge of social justice issues alongside educating the public. The advocator shares information regarding specific laws and policies related to social justice, such as sharing petitions to introduce psychologically grounded laws that reduce disparities in marginalised communities. The agitator raises information on social justice issues, with a focus on controversial topics aimed at challenging opinions. This could include sharing links to news articles that inform readers on controversial topics to try to reduce the echo chamber of social media opinions. Finally, the supporter uses social media to support social justice causes either psychologically, such as signing petitions and sharing therapeutic resources, or financially, through donations. Therefore, even if psychologists do not want to become mental health influencers, they can still use social media for social justice advocacy on a smaller scale by adopting some of these other roles, for example, sharing posts in the informer role to their friends and family from their personal social media account.

### Mental health literacy

The psychologists in this study recognised the power of sharing simple psychoeducation resources. Access to these resources could support people to care for their mental wellbeing and may be the first step into accessing therapy, through facilitating recognition of their psychological distress. Lily reflected on how many therapy sessions begin with psychoeducation and so providing these resources can act as a first step before beginning therapy as ‘if they've already got that, that can make a difference’. Multiple psychologists referenced their preference for sharing simple cognitive behavioural therapy (CBT) resources, such as a post explaining that thoughts are not facts. These more generalised posts could support individuals who have not accessed therapy previously and introduce them to CBT offered by the NHS. This fits with prominent mental health influencer and clinical psychologist Dr. Julie Smith's description in a podcast interview of how she initially identified her motivation to begin her Instagram and TikTok accounts. Smith recognised the impact on her clients of sharing psychoeducation in therapy and believed these tools should be accessible to people outside of therapy: ‘they were so raring to go and so sort of empowered by having their own tools to use day in and day out to impact on how they felt. You know it just felt like why should people have to pay to come to see people like me to find out that educational stuff? You know I might not be able to give therapy to everybody, but the educational stuff can be shared as widely as possible’ (Wallace, 2022).

Moreover, this fits with the psychologists in this study acknowledging that therapy can be difficult to access and expensive, with frustrations at the long waiting lists people face to access mental health support. Therefore psychologists could use Instagram for social justice advocacy by sharing free content to increase accessibility to psychoeducation. Mitch recognised ‘it needs to be about sort of breaking down the mystery around psychology, it needs to be about sort of reducing the stigma around mental health needs, it needs to be making information more accessible’. This suggests this knowledge‐sharing serves a dual purpose both of educating people around mental health who do not have access to these resources and of reducing stigma. The accessibility of psychoeducation via social media may be particularly valuable for people from cultures who still consider discussing mental health or therapy as taboo subjects and so may be less likely to buy self‐help books or access therapy (Matei, 2019). Indeed, Office for National Statistics (2022) statistics reported that the Asian ethnic group was statistically underrepresented in IAPT services, with 6.5% of individuals who identified as from an Asian ethnic group with a potential common mental disorder receiving treatment from IAPT services compared to 9.7% of individuals who identified as from a White ethnic group. These individuals could be introduced to simple psychoeducation for free on Instagram, which may contribute to increased mental health knowledge and reduced stigma so the individual feels more capable of accessing mental health support (Naslund et al., 2016). Online help‐seeking behaviour appears to act as a gateway to accessing professional mental health support, as individuals have the opportunity to use the mental health information they access online to inform their decision of if they need support (Pretorius et al., 2019). Moreover, individuals may feel empowered through sharing their mental health issues online with other people with similar experiences creating a sense of belonging and shared identity (van Uden‐Kraan et al., 2009). This online self‐disclosure in turn can facilitate individuals feeling more confident in disclosing their mental health issues face to face, such as to a health professional or family member (McKenna & Bargh, 1998). Therefore, psychoeducation posts can act as a catalyst for change with individuals feeling more informed and confident to self‐disclose about their mental health and access support.

The psychologists in this study voiced their concerns about striking the delicate balance between sharing detailed evidence‐based psychoeducation that they believed would actually be useful in increasing mental health literacy and meeting the formatting requirements for the algorithm to show their posts for the public to engage with. This echoes the subthemes ‘we have to reclaim that this stuff is complex’ and ‘a lot of it is pressure to perform and I'm not a performer’ (White & Hanley, 2025). Grace acknowledged that content may be oversimplified to the point of being incorrect, sharing ‘I think the risk is that if you're trying to make something that is actually very complex into something really simple so people want to take away sound bites, so “if I procrastinate it's because I experienced trauma” or “I can't do this because of my attachment style”’. This is supported by clinical psychologist Dr. Julie Smith questioning in a podcast interview ‘how can I talk about quite possibly the least sexy subject there is and hold people's attention?’ so that people did not scroll past the psychoeducation posts she had invested time and energy into creating (Wallace, 2022). Medina and Montell (2022) noted the current societal tendency to make information as quick and easy to consume as possible, exacerbated by social media. However, they question if highly complex topics around mental health should be made ‘digestible’ in this way. Medina and Montell (2022) also observed that the largest mental health influencer accounts are focused on broad and open‐to‐interpretation topics, such as trauma and anxiety, rather than more specific mental health disorders such as obsessive‐compulsive disorder (OCD) and personality disorders. Mia reflected that psychologists typically focused more on anxiety and depression as ‘more palatable signs of mental health’, rather than suicide and chronic mental health conditions, creating a ‘glamorisation of mental health’ and thus potentially contributing to mental health stigma for what can and cannot be discussed. Therefore, it appears that currently, mental health influencers may be increasing the public's mental health literacy of certain types of psychological distress that already have less stigma, such as sharing posts about anxiety rather than personality disorders.

Pretorius et al. (2022) used deductive content analysis to understand the type of content shared by the largest mental health professional accounts on Instagram and TikTok. However, a limitation is the study only analysed the most recent five posts by each account. Results showed that 66.43% of the mental health professionals' Instagram posts were for the general purpose of education. The results were then further categorised into the focus of the education posts, with 31.43% about personal growth and 28.18% about the promotion of a service, event or product. Considering mental health literacy, the Instagram posts shared information on self‐help strategies, knowledge about professional support available and encouraged appropriate help‐seeking. The posts also taught people how to recognise symptoms of specific disorders and to identify risk factors. Although none of the psychologists in the present study explicitly used the phrase mental health literacy, their posts fulfilled the same criteria as Pretorius et al. (2022) in increasing mental health literacy, such as sharing information on different types of distress. Jorm et al. (1997) define mental health literacy as ‘knowledge and beliefs about mental disorders which aid their recognition, management and prevention’. Mental health literacy can be divided into six components: ‘(a) the ability to recognise specific disorders or different types of psychological distress; (b) knowledge and beliefs about risk factors and causes; (c) knowledge and beliefs about self‐help interventions; (d) knowledge and beliefs about professional help available; (e) attitudes which facilitate recognition and appropriate help‐seeking; and (f) knowledge of how to seek mental health information’. Jorm (2000) suggested people gain most of their knowledge about mental health from personal experiences and family and friend's experiences. The study added that media, such as television and the internet, also influence public perception of mental health. However, this study was published prior to social media, which arguably holds one of the largest influences over public perceptions of mental health in the present day. Psychologist use of Instagram can be seen as social justice advocacy through increasing mental health literacy as posts can fulfil each of the six components outlined by Jorm et al. (1997).

Interestingly, none of the purposes for social media use identified by Pretorius et al. (2022) were for social justice, which stands in stark contrast to multiple participants in the present study identifying social justice as their primary motivation for their accounts. However, through increasing mental health literacy and making psychoeducation more accessible, many of these posts could be categorised as a form of social justice work. Pretorius et al. (2022) found that although mental health professionals posted a similar number of posts about psychoeducation on TikTok (67.86%) and Instagram (66.43%), the focus of the education posts differed on each platform. The psychoeducation posts on TikTok had a greater focus on mental health concerns, with 30.00% teaching people to recognise specific disorders. Additionally, 20.00% of TikTok education posts were for the purpose of increasing engagement with the audience, often through entertaining posts to gain followers and possibly promote their services. Only 2.86% of mental health influencer's posts on TikTok were for promotion, compared to 28.18% on Instagram. This suggests that at the time of the study, TikTok was used less for promoting private therapy services. This fits with this study, with multiple psychologists using their Instagram accounts from a business perspective to promote themselves as therapists to gain private clients or to advertise passive products such as online courses and books. Therefore, consideration needs to be taken of how psychologists may use different social media platforms in alternative ways when creating ethical guidelines, rather than blanket guidelines across all platforms.

### Strengths, limitations and areas for future research

The study is the first qualitative research to discuss how Instagram is currently being used for social justice advocacy by psychologists working as mental health influencers. This paper used a social justice lens to focus in greater depth on the uses and issues around social justice advocacy as a mental health influencer. Although this study interviewed UK‐based psychologists who discussed current systemic issues in the United Kingdom, these findings are still applicable to other countries with similar systemic issues, such as racism and sexism. Furthermore, this study is the first to draw attention to the impact of systemic issues on social media use as a mental health influencer. This was an unexpected contribution to knowledge, as previous literature has not examined the role of systemic issues on psychologists' motivations to use social media as a mental health influencer.

Future qualitative research could interview psychologists who hold social media accounts as mental health influencers to further explore how they use social media for social justice advocacy. Questions could consider how the five different roles that Al'Uqdah et al. (2019) conceptualised for social justice work by psychologists on social media are put into practice. Although this study has a large focus on how social media can be used for social justice advocacy, this was not a primary focus of the broader research project, and so is not reflected in the interview schedule or the participant information sheet. Different psychologists may come forwards for an interview on social media use for social justice advocacy, compared to the original recruitment for an interview on ethical guidelines for psychologist social media use.

## IMPLICATIONS AND CONCLUSIONS

The findings of this study emphasise the potential use of social media by psychologists for social justice advocacy. Winter (2019) encourages psychologists to consider broader resources that individuals can use to support their mental health, as a way to bring a social justice perspective to their work. Psychologists can focus on the uniqueness of the individual, to consider if individual therapy is the most helpful option, or if they can access support from their broader community. This definition of resources from the broader community could be extended to include the online community of resources from mental health influencers. Therefore, psychologists could adopt a social justice perspective on social media in two ways: firstly, as described throughout this study by setting up their own social media account as a mental health influencer to disseminate psychoeducation and raise public consciousness of systemic issues contributing to their psychological distress; and secondly, and potentially more appealing to many due to the lower workload, to share resources created by psychologists with clients and other staff in an informer role from their personal social media account (Al'Uqdah et al., 2019). A few psychologists described how they would sometimes bring in useful psychoeducation drawings they found on Instagram to explain relevant concepts to clients in therapy or to train staff. Within formulations, psychologists often consider protective factors that help the client cope and manage stressors with a focus on building up self‐care strategies within sessions (Dudley & Kuyken, 2014). Psychologists could recommend following mental health influencers as a way to access ongoing psychoeducation and self‐care guidance after therapy ends. This may be particularly beneficial to young people, with 89% of 16 to 24 year olds using social media to support their wellbeing (Ofcom, 2023). Trainee psychologists could be taught of the five roles conceptualised by Al'Uqdah et al. (2019) for how psychologists can incorporate social justice work into their practice through social media. Therefore, even if psychologists choose not to become mental health influencers, they could still utilise their personal social media accounts for social justice, such as sharing information about a protest in the informer role or making an online donation in the supporter role.

A key recommendation is for ethical guidance on psychologists' social media use to acknowledge the use of social media for social justice advocacy and the related ethical issues psychologists may encounter. The guidance can support psychologists with how to combine the roles of a psychologist and a businessperson to market their services on social media, acknowledging the potential contradictions between these two identities. For example, influencers are encouraged to engage with followers through messages to promote products and to put passive products behind paywalls, which may feel incompatible with the boundaries and values of a psychologist. Whilst testimonials are often used to grow businesses, psychologists should gain consent from clients to share these on social media, with a discussion of how the client would feel if others saw this and the power dynamic between the client and psychologist. Client confidentiality must be prioritised, with any testimonials anonymised. This study recommends that psychologists consider the power their professional title holds, which could influence the public to purchase any products they promote. The psychologist should consider what types of products they could ethically promote through a sponsored post, if any, and if they have the competency to promote this. Whilst psychologists may be asked by the media to discuss issues outside of their area of expertise, which could raise their public profile, they should only comment on issues within their competency.

Jorm (2000) suggests increasing mental health literacy could lead to earlier interventions and prevention of psychological distress, in addition to the effectual use of self‐help strategies and community support. Psychologists' use of social media as a mental health influencer to provide psychoeducation based on their professional training and experiences could increase mental health literacy, so the public can learn self‐care strategies to manage their distress. This increased literacy would enable people to better recognise psychological distress at earlier stages to then receive interventions that require lower levels of therapeutic support. These two factors in turn could help to reduce the strain on mental health services, and the pressure felt by psychologists within these services to support a high number of clients. A few psychologists in this study set up Instagram accounts for the mental health service where they worked to share resources with clients who accessed their service. Whilst most psychologists work in private practice, meaning their Instagram account could be perceived as a business profile to gain clients, NHS or third‐sector services could also benefit from an Instagram account to share resources. Therefore, irrespective of working in the public or private sector, there is an opportunity for psychologists to incorporate social media into their work.

## AUTHOR CONTRIBUTIONS

**Ella White:** Conceptualization; writing – original draft; methodology; writing – review and editing; formal analysis; investigation; visualization; project administration; resources. **Terry Hanley:** Supervision; writing – review and editing.

## CONFLICT OF INTEREST STATEMENT

The authors declare no conflicts of interest.

## Data Availability

The data that support the findings of this study are available from the corresponding author upon reasonable request.
